# Development of a cross-cultural scale on attitudes toward gender and sexual diversity (AGSD)

**DOI:** 10.3389/fsoc.2025.1510002

**Published:** 2025-01-31

**Authors:** Daniel Oleas, Xochitl Garza-Olivares, Fernando Silva Teixeira-Filho, Guido Mascialino, Jose A. Rodas

**Affiliations:** ^1^Department of Psychology, Ecotec University, Samborondón, Ecuador; ^2^Department of Clinical and Health Psychology, Tecnológico de Monterrey, Monterrey, Mexico; ^3^Department of Clinical Psychology, Universidade Estadual Paulista, Assis, Brazil; ^4^Escuela de Psicología y Educación, Universidad de Las Américas, Quito, Ecuador; ^5^Escuela de Psicología, Universidad Espíritu Santo, Samborondón, Ecuador; ^6^School of Psychology, University College Dublin, Dublin, Ireland

**Keywords:** gender and sexual diversity, scale validation, Ecuador, factor analysis, Latin America

## Abstract

**Background:**

Attitudes toward gender and sexual diversity can range from acceptance to rejection, influenced by various social, psychological, and cultural factors. In Latin America, instruments tailored to measure these attitudes within specific cultural contexts are limited. This study aimed to develop and validate a culturally relevant scale to assess attitudes toward gender and sexual diversity in Ecuador.

**Methods:**

The research was conducted in two studies. In Study 1, an exploratory factor analysis (EFA) was performed on data collected from 225 psychology students to identify the scale's structure. In Study 2, a confirmatory factor analysis (CFA) was conducted with 362 students to confirm the factor structure and assess the scale's validity. The final scale comprised 18 items across three factors: social coexistence, moral and pathological views, and stereotypes.

**Results:**

The scale demonstrated sound psychometric properties, with acceptable internal consistency (Cronbach's α = 0.74–0.77). Factor loadings ranged from 0.56 to 0.87, confirming the robustness of the scale. Three distinct factors were identified, providing a comprehensive measure of attitudes toward gender and sexual diversity in social, psychological, and behavioral contexts.

**Conclusion:**

This scale represents a valuable tool for assessing attitudes toward gender and sexual diversity in Latin American populations. Future research should test its applicability across broader populations and in different Latin American countries to further validate its use and generalizability.

## Introduction

### Attitudes toward gender and sexual diversity

Attitudes toward gender and sexual diversity encompass a broad range of beliefs, perceptions, and behaviors, that may be both positive and negative. These attitudes can significantly influence social and psychological dynamics, shaping how individuals who do not experience sexuality in a traditional manner are perceived and treated within various societal contexts. Studies in this field are relatively recent, with homosexuality being one of the first forms to be investigated. Initially, the concept of homophobia, coined by clinical psychologist George Weinberg in 1972, referred to the rejection or irrational fear of homosexual individuals, leading to stigmatization and social exclusion. Such negative attitudes have profound effects, contributing to discrimination that limits opportunities and adversely impacts the psychological well-being of LGBT+ individuals (Weinberg, 1972; Junqueira, 2012).

In recent decades, there has been increasing recognition of non-normative gender identities and sexual orientations, such as those of bisexual, transgender, non-binary or gender fluid. Despite this progress, research on attitudes toward these identities remains insufficient. While there has been substantial research into homophobia, the attitudes directed at non-cisgender and non-heterosexual individuals, such as transphobia and biphobia, have not been explored as thoroughly in academic literature. This gap highlights the need to broaden research to more comprehensively examine attitudes toward the broader LGBT+ community, recognizing the diversity of identities and experiences within it.

### Evaluating attitudes toward gender and sexual diversity

Evaluating these attitudes is essential for understanding and addressing discrimination. By assessing the prevalence and underlying factors of both positive and negative attitudes, researchers and practitioners can develop targeted interventions that promote inclusivity and reduce stigma in academic, professional, and social settings. Such evaluations offer valuable insights into how these attitudes are internalized and perpetuated, especially among key groups such as psychology students, who will become future mental health professionals. Given their ethical responsibility to promote equality and uphold human dignity, it is crucial to equip these students with the tools needed to challenge biases and foster an environment of acceptance and respect (Hailes et al., 2021; Korkut, 2020).

Despite the importance of assessing attitudes toward gender and sexual diversity, many of the existing measurement instruments present several limitations. Numerous scales primarily focus on homophobia, often neglecting the experiences and identities of non-cisgender and non-heterosexual individuals, such as transgender and non-binary people. As a result, these tools may fail to capture the full spectrum of attitudes toward the LGBT+ community. Moreover, most of these instruments have been developed in English-speaking countries and reflect the cultural contexts of those regions, which may not align with the social realities of other parts of the world, particularly Latin America (Maron and Rondini, 2013).

The limited availability of scales adapted for Latin American contexts is a significant gap in the literature. Research shows that among the instruments developed to assess attitudes toward gender and sexual diversity, only a small number have been validated for use in Latin America (see Table 1 for a description of these scales: Quiles del Castillo et al., 2003; Marinho et al., 2004; Costa et al., 2015; De Miranda Ramos and Cerqueira-Santos, 2020). Additionally, many of these scales focus on specific dimensions, such as homophobia, and are often designed for educational contexts aimed at reducing homophobic bullying. Few instruments consider the broader spectrum of LGBT+ identities or the complex, intersectional forms of discrimination that people face.

**Table 1:** Attitudes toward gender and sexual diversity scales in 2021.

**Name**	**Authors**	**Dimensions**	**Sample**	**Internal consistency**
Open and subtle homophobia scale	Quiles del Castillo et al., 2003	Open homophobia and subtle homophobia.	Psychology students	0.78 subtle homophobia. 0.84 open homophobia.
Adaptation of the Implicit and Explicit Homophobia Scale to the Brazilian context.	Marinho et al., 2004	Implicit homophobia and explicit homophobia.	University students	0.87 general
Gender and Transphobia Scale	Hill and Willoughby, 2005	Gender, Transphobia, and Gender Discrimination	University students	0.91 general; 0.83 gender; 0.94 transphobia; 0.79 gender discrimination
Validation of prejudice against sexual and gender diversity.	Costa et al., 2015	Genderism-transphobia and gender violence	University students, teachers, and staff	0.96 general
Scale of beliefs and attitudes about homosexuality	Yertutanol et al., 2019	Heterosexism, homophobia, homonegativity, and neutrality.	University students, teachers, and staff	0.85 heterosexism; 0.95 homophobia; 0.95 homonegativity; 0.086 neutrality
Scale of attitudes toward homosexuality	De Miranda Ramos and Cerqueira-Santos, 2020	Distal attitudes and proximal attitudes	Brazilian individuals over the age of 18	0.85 distal attitudes; 0.81 proximal attitudes
Adaptation of the Scale of Teachers' Attitudes and Beliefs about Homosexuality Scale (EACPH)	Rondini et al., 2021	Open homophobia and subtle homophobia.	Teachers	Adaptation shows content validation

This highlights the need for culturally relevant tools that can accurately measure these prejudices within the specific sociocultural dynamics of Latin American countries. Without such tools, efforts to reduce LGBT+ phobia and promote inclusivity may fall short of addressing the full range of discriminatory attitudes present in these societies.

### The current study

In light of the gaps identified in the evaluation of attitudes toward gender and sexual diversity, particularly in Latin American contexts, this research aims to address the need for a culturally relevant instrument. The present article reports the results of two studies conducted to develop and validate a scale assessing attitudes toward this form of diversity. The first study focused on the creation of the scale by generating items based on theoretical dimensions of attitudes toward gender and sexual diversity and conducting an exploratory factor analysis (EFA) to explore its structure. The second study involved a confirmatory factor analysis (CFA) using a separate subsample to assess the scale's validity and reliability. Together, these studies ensured that the instrument accurately measures attitudes toward gender and sexual diversity within the target population.

## Methods

Two studies were conducted to develop and validate a scale measuring attitudes toward gender and sexual diversity. In the first study, an EFA was performed to identify the underlying structure of the scale and to refine the item pool. The initial item pool, developed through expert review and theoretical considerations, was administered to a sample of psychology students. Based on these results, a set of 18 items was selected for further testing. In the second study, a CFA was conducted to evaluate the scale's validity and reliability. The CFA used a different sample of psychology students and evaluated the robustness of the three-factor model.

For more information on the methodology, database, and scale items, please refer to the Open Science Framework pre-registration at: https://osf.io/2cj85/.

### Study 1

#### Participants

The first sample consisted of 225 psychology students from Ecotec University in Ecuador. Participants were categorized into the following age groups: 155 students (68.89%) were born between 2000 and 2005, 52 students (23.11%) between 1995 and 1999, 10 students (4.44%) between 1990 and 1994, 6 students (2.67%) between 1985 and 1989, and 2 students (0.89%) between 1980 and 1984.

Regarding gender identity, 68.44% (*n* = 154) identified as cisgender women, 26.22% (*n* = 59) as cisgender men, 4.89% (*n* = 11) preferred not to disclose, and 0.44% (*n* = 1) identified as non-binary. In terms of ethnicity, 91.11% self-identified as mestizo, 6.67% as white, 0.44% as indigenous, and 0.89% chose not to specify their ethnic background. The participants ranged from first-year to fifth-year students, with the largest proportion being first-year students (30.22%) and the smallest in their fifth year (8%).

#### Procedure and instruments

The conceptual dimensions of the scale were established using the model proposed by Yertutanol et al. (2019). The items were adapted to assess attitudes toward LGBT+ individuals, with additional items designed, resulting in a total of 47 statements. These statements were written in the first person and formulated in neutral language to ensure comprehension across different Latin American countries.

The item pool was reviewed by six expert judges from Brazil, Ecuador, and Mexico, all of whom had experience in scale development or gender theory. The number of judges followed the recommendations of Hyrkäs et al. (2010). Each judge received the initial draft of 47 items, along with an explanation of the scale's objective, the definitions of each theoretical dimension, and instructions to evaluate whether the items were appropriate for measuring attitudes and suitable for the target population (DeVellis, 2017). Following the review and corrections from the judges, four items were removed due to issues with neutral language. A four-point Likert scale, ranging from “strongly disagree” to “strongly agree,” was used to assess participants' responses, promoting more decisive answers by eliminating a neutral option, simplifying interpretation, and potentially improving data reliability (Croasmun and Ostrom, 2011).

The final version of the scale, which also included sociodemographic information, was administered between January 2023 and January 2024. Data collection was conducted through an electronic Google Forms questionnaire distributed via email and WhatsApp. Before completing the survey, participants provided electronic consent. Confidentiality and anonymity were maintained throughout the process.

The data were analyzed using Factor software (Lorenzo-Seva and Ferrando, 2023) for preliminary item reduction, descriptive analysis, and EFA.

#### Data analysis

A preliminary analysis of the items was conducted with the first sample using Gulliksen's pool (Ferrando et al., 2023), aiming to address potential challenges in assessing the dimensionality and structure of the scale. The goal of this pre-analysis was to identify and eliminate items based on their extremity and consistency, evaluated through the Relative Difficulty Index (RDI) and Item Consistency Index (ICI) (Lorenzo-Seva and Ferrando, 2021).

Descriptive statistics were calculated for each item, including the mean (M), standard deviation (SD), skewness, and kurtosis. The assumption of normality was tested using the skewness and kurtosis indices, with values between ±1.5 considered within normal thresholds (Cuadras, 2016).

EFA was then performed on the first sample using the robust unweighted least squares (RULS) estimator due to the ordinal nature of the items (Muthen and Kaplan, 1992). It is also important to emphasize that the sample size used in the present study is sufficient for robust factor analysis, as it exceeds the recommended minimum threshold of 5–10 participants per item (Hogarty et al., 2005). This recommendation aligns with broader methodological guidelines that underscore the importance of sizable samples to ensure reliable and generalizable factor-analytic solutions. Otherwise model fit was evaluated using the chi-square test (χ^2^) and the root mean square error of approximation (RMSEA), with values between 0.05 and 0.08 indicating an acceptable fit (Kline, 2016). Additionally, other fit indices, including the comparative fit index (CFI), goodness-of-fit index (GFI), and Tucker-Lewis index (TLI), were used to assess model fit, with values above 0.90 considered acceptable (Schumacker and Lomax, 2015).

### Study 2

#### Participants

The second sample included 362 students from the same Psychology program. The average age was 21.41 (SD = 3.34). About 75.41% (*n* = 273) identified themselves as cisgender women, 18.23% (*n* = 66) as cisgender men, 2.76% (*n* = 10) preferred not to respond, 2.76% (*n* = 10) identified themselves as non-binary, and 0.83% (*n* = 3) as transgender. About 333 (91.99%) self-defined as mestizo, 16 as white, 5 preferred not to answer, 3 as black, 1 as mulatto, 1 as indigenous and 3 self-defined as montubio. Regarding sexual orientation, the data revealed that 76.52% (*n* = 277) of the participants identified as heterosexual. Other orientations included 14.09% (*n* = 51) identifying as bisexual, 3.31% (*n* = 12) as pansexual, 1.93% (*n* = 7) as homosexual, 2.49% (*n* = 9) as other, 1.38% (*n* = 5) who were unsure how to respond, and 0.28% (*n* = 1) identifying as asexual.

#### Procedure and instruments

In the second study, a refined set of 18 items, which emerged from the EFA in the initial study, was used. Participants were asked to rate on a four-point Likert scale ranging from 1 (completely disagree) to 4 (completely agree). This analysis aimed to confirm the robustness and validity of the scale's dimensionality and structure. The scale, which also included sociodemographic information, was distributed between February 2023 and October 2024 in two Ecuadorian universities. Data were collected through an electronic questionnaire hosted on Google Forms and shared via email and classroom visits promoting the study. Participants provided electronic consent before completing the survey. Throughout the process, researchers ensured strict confidentiality and maintained participants' anonymity. For CFA and reliability testing, the JASP software (JASP Team, 2023) was used.

#### Data analysis

For the second sample, descriptive statistics for the items were first computed. Then, a CFA was conducted using the Diagonally Weighted Least Square (DWLS) estimator, appropriate for the ordinal nature of the data. The model fit was assessed using the same goodness-of-fit indices as in the EFA. Factor loadings (λ) >0.40 were considered satisfactory. This estimation strategy, are strongly advised because they minimize biases that can occur with Maximum Likelihood (ML) when handling ordinal data without a strict assumption of multivariate normality. DWLS uses polychoric correlations instead of covariances, thereby improving parameter estimates and goodness-of-fit measures for Likert-type instruments, even when the variables are not normally distributed. Moreover, as long as a sufficiently large sample is available (*n* > 200), DWLS can secure more robust validity evidence, irrespective of how many response categories each item contains (Li, 2016).

Finally, the internal consistency of the scale was evaluated using both Cronbach's alpha (α; Cronbach, 1951) and the omega coefficient (ω; McDonald, 2013), with values between ω = 0.60 and ω = 0.80 considered acceptable (Raykov and Hancock, 2005).

## Results

### Study 1

#### Item reduction

The analysis using the RDI and ICI resulted in the removal of 23 items that did not meet the efficacy criteria. Additionally, the Measure of Sampling Adequacy (MSA) at the item level indicated the need to eliminate two more items. As a result, 18 items were retained for further analysis. Detailed information about the pre-factor analysis can be accessed online (https://osf.io/q7hdk).

#### Descriptive analysis of the items and exploratory factor analysis

Table 2 provides the descriptive statistics for each item and the results of the exploratory factor analysis. Items 3, 10, 12, and 18 had higher means compared to the others, while items 10, 14, and 18 showed the highest variability. Skewness (g1) and kurtosis (g2) values revealed significant deviations from normality in 7 out of the 18 items, specifically items 5, 7, 8, 9, 12, 13, and 16.

**Table 2:** Descriptive statistics and exploratory factor analysis from Study 1.

**Item**	**Mean(SD)**	**g1**	**g2**	**f1**	**f2**	**f3**
1. Sexual perversions are more common in homosexual men than in heterosexual men [Las perversiones sexuales son más comunes en hombres homosexuales que en heterosexuales].	2.02 (0.98)	0.51	−0.85	0.12	0.02	**0.70**
2. I feel some distrust toward LGBT+ people [Me dan cierta desconfianza las personas LGBT+].	1.51 (0.79)	1.49	1.46	−0.19	0.27	**0.41**
3. Organizations that defend the rights of LGBT+ people are necessary [Las organizaciones que defienden los derechos de las personas LGBT+ son necesarias].	3.34 (0.84)	−1.17	0.69	**0.67**	−0.10	0.01
4. Homosexual men are less masculine than heterosexual men [Los hombres homosexuales son menos masculinos que los heterosexuales].	2.11 (0.99)	0.39	−0.98	0.01	**0.34**	0.24
5. I would hit an LGBT+ person if they flirt with me [Golpearía a una persona LGBT+ si intenta coquetearme/ligarme].	1.28 (0.65)	2.54	6.35	−0.26	0.19	**0.34**
6. LGBT+ people have more sexual desire than heterosexual people [Las personas LGBT+ tienen más deseo sexual que las personas heterosexuales].	1.54 (0.81)	1.32	0.75	0.07	–**0.30**	**1.03**
7. LGBT+ people talk about sex all the time [Las personas LGBT+ hablan todo el tiempo sobre sexo].	1.42 (0.72)	1.82	2.96	−0.04	−0.01	**0.75**
8. People should have the right to choose whether or not to have an LGBT+ neighbor [Las personas deberían de tener el derecho de escoger tener o no un vecino/a LGBT+].	1.35 (0.78)	2.29	4.36	0.03	**0.40**	**0.42**
9. Every police station should identify the LGBT+ individuals in their area [Cada estación de policía debería tener identificados a los LGBT+ de su área].	1.42 (0.76)	1.86	2.71	0.15	**0.37**	**0.44**
10. I don't care if I go to LGBT+ bars, clubs, or parties [Me da igual si voy a bares, clubes o fiestas LGBT+].	3.02 (1.10)	−0.70	−0.91	**0.79**	0.13	0.03
11. Children from dysfunctional families are more likely to be LGBT+ [Los niños y niñas de familias disfuncionales son más propensos a ser LGBT+].	1.54 (0.83)	1.45	1.19	0.18	**0.76**	0.14
12. I don't mind seeing an LGBT+ couple together in public [Me da igual ver una pareja LGBT+ junta en público].	3.46 (0.99)	−1.68	1.39	**0.78**	0.04	0.09
13. Lesbians have less sexual desire than heterosexual women [Las lesbianas tienen menos deseo sexual que las mujeres heterosexuales].	1.36 (0.67)	2.07	4.32	−0.07	−0.07	**0.78**
14. The media and social networks promote homosexuality [Los medios de comunicación y redes sociales difunden la homosexualidad].	2.28 (1.06)	0.13	−1.28	0.04	**0.87**	−0.26
15. The transgender condition should remain classified as a mental disorder [La condición transgénero debería de mantenerse como un trastorno mental].	1.61 (0.94)	1.38	0.69	–**0.30**	**0.59**	−0.07
16. An LGBT+ person should not be allowed to hold high-level government positions [Una persona LGBT+ no debería tener permiso para ocupar cargos de alto nivel en el gobierno].	1.45 (0.84)	1.86	2.51	−0.12	0.24	**0.49**
17. Despite their morals and job opportunities, LGBT+ people are more likely to engage in prostitution [A pesar de su moral y las oportunidades de trabajo, las personas LGBT+ son más propensas a prostituirse].	1.72 (0.90)	0.93	−0.33	−0.10	0.15	**0.45**
18. I don't care if my friends are LGBT+ or heterosexual [Me da igual si mis amigos/as son LGBT+ o heterosexuales].	3.40 (1.06)	−1.47	0.53	**0.62**	0.09	−0.12

In bold Significant factor loading; g1, skewness; g2, kurtosis.

The factorial adequacy tests indicated that the polychoric correlation matrix was suitable for EFA, with a KMO value of 0.87. Bartlett's Test of Sphericity also confirmed the applicability of EFA, with a value of 2200.2 (*df* = 153; *p* < 0.001). Additionally, the chi-square test (χ^2^ = 146.54, *df* = 102; *p* < 0.001) and the root mean square error of approximation (RMSEA = 0.044) demonstrated a good model fit. The RULS extraction method identified three factors that explained 58.72% of the variance, indicating that there was no need to eliminate any items. Furthermore, the model fit indices—CFI = 0.992, GFI = 0.984, and TLI = 0.987—suggest that the three-dimensional model provided an adequate fit for the total sample of participants.

### Study 2

#### Descriptive analysis of the items

Table 3 presents the descriptive statistics for each item. Items 3 and 12 had the highest means, while items 10 and 14 showed the greatest variability. Skewness (g1) and kurtosis (g2) indicated clear deviations from normality in 5 out of the 18 items, specifically items 5, 8, 9, 16, and 18.

**Table 3:** Descriptive analysis from Study 2.

**Item**	**Mean**	**SD**	**g1**	**g2**
Item 1	2.25	0.97	0.11	−1.08
Item 2	1.66	0.86	1.09	0.22
Item 3	3.23	0.91	−1.02	0.18
Item 4	2.20	0.99	0.29	−1.01
Item 5	1.37	0.75	2.18	4.12
Item 6	1.65	0.87	1.13	0.29
Item 7	1.47	0.74	1.49	1.43
Item 8	1.49	0.81	1.57	1.58
Item 9	1.46	0.80	1.61	1.54
Item 10	2.90	1.15	−0.62	−1.08
Item 11	1.74	0.94	0.94	−0.34
Item 12	3.38	0.93	−1.36	0.73
Item 13	1.54	0.75	1.34	1.31
Item 14	2.33	1.00	0.12	−1.09
Item 15	1.68	0.95	1.10	−0.04
Item 16	1.51	0.85	1.52	1.16
Item 17	1.85	0.92	0.68	−0.68
Item 18	3.61	0.79	−2.19	4.03

g1, skewness; g2, kurtosis.

#### Confirmatory factor analysis (CFA)

In the second study, we proposed a different item structure from that identified by the EFA in Study 1. This new structure was based on the content of the items, as the previous grouping did not consider this aspect. We analyzed the new structure using CFA with DWLS estimation. Item 16 was dropped because it did not fit with any of the three groups, and items 2, 5, 8, and 9 were reverse scored. The results (χ^2^_(281)_ = [*p* < 0.001]) and other fit indices, including RMSEA, SRMR, CFI, and TLI, indicated a good model fit: RMSEA = 0.06, SRMR = 0.08, CFI = 0.99, and TLI = 0.98. Factor loadings for each item were high and significant, ranging from 0.56 to 0.87 (see Table 4).

**Table 4:** Confirmatory Factor Analysis: factor loading.

						**95% Confidence interval**	
**Factor**	**Item**	**Estimate**	**Std. error**	***z*-value**	***p***	**Lower**	**Upper**
Social coexistence	3	0.62	0.02	29.35	< 0.001	0.57	0.66
	10	0.56	0.02	25.16	< 0.001	0.51	0.60
	12	0.61	0.02	28.88	< 0.001	0.56	0.65
	18	0.61	0.02	28.28	< 0.001	0.57	0.65
	2	0.84	0.02	42.81	< 0.001	0.80	0.87
	5	0.73	0.02	31.57	< 0.001	0.68	0.77
	8	0.81	0.02	39.56	< 0.001	0.77	0.85
	9	0.79	0.02	36.44	< 0.001	0.75	0.83
Moral and pathological views	1	0.62	0.02	30.11	< 0.001	0.58	0.66
	11	0.70	0.02	34.18	< 0.001	0.66	0.74
	14	0.62	0.02	28.83	< 0.001	0.58	0.66
	15	0.86	0.02	40.10	< 0.001	0.82	0.90
	17	0.66	0.02	31.40	< 0.001	0.61	0.70
Stereotypes	4	0.61	0.02	26.78	< 0.001	0.57	0.66
	6	0.79	0.02	39.88	< 0.001	0.75	0.83
	7	0.87	0.02	39.41	< 0.001	0.83	0.91
	13	0.79	0.02	38.41	< 0.001	0.75	0.83

#### Internal consistency

Internal consistency of all factors was calculated using Cronbach's alpha to facilitate comparison with other future studies and McDonald's omega (ω), which is a less biased alternative (Trizano-Hermosilla and Alvarado, 2016).

As observed in Table 5, the three factors presented acceptable to excellent reliability, with alphas of 0.74 to 0.77, respectively.

**Table 5:** Internal consistency.

**Factor**	**McDonald's ω**	**Cronbach's α**
1	0.75	0.77
2	0.75	0.75
3	0.74	0.74
Total	0.88	0.88

## Discussion

The primary aim of this study was to develop and validate a culturally relevant scale to measure attitudes toward gender and sexual diversity. This process involved the creation of items, expert review, and statistical analysis, and the results from both the EFA and CFA indicate that the scale is a reliable and valid tool. The three-factor structure—encompassing Social Coexistence, Moral and Pathological Views, and Stereotypes—demonstrates the complexity of attitudes that people hold toward LGBT+ individuals, highlighting the scale's ability to capture a broad spectrum of beliefs and attitudes that can be either positive or negative.

The first factor, Social Coexistence, captures the degree to which individuals are comfortable interacting with LGBT+ people in everyday social situations. This factor includes items addressing comfort with LGBT+ visibility in public spaces, such as bars and social settings, and with LGBT+ friends or acquaintances. Low scores in this factor indicate discomfort or reluctance to engage with LGBT+ individuals in social contexts, while higher scores suggest more accepting and inclusive attitudes.

Factor 2, Moral and Pathological Views, evaluates beliefs that conceptualize LGBT+ identities as associated with certain psychological or behavioral characteristics. This factor includes items related to the idea that being transgender should be classified as a mental disorder (item 14) and the belief that homosexual men are more likely to engage in sexual perversions than heterosexual men (item 1). It also includes perceptions that LGBT+ individuals may have certain social disadvantages or negative behavioral tendencies, such as the belief that children from dysfunctional families are more likely to be LGBT+ (item 11), or that LGBT+ people are more likely to engage in prostitution (item 17). Additionally, this factor assesses views regarding the appropriateness of LGBT+ individuals holding positions of authority (item 15). Respondents scoring higher on this scale tend to view LGBT+ individuals as suffering from behavioral, emotional, and sexual disorders or deficiencies, and are more likely to perceive them as socially and morally unfit for positions of authority or leadership.

Factor 3, Stereotypes, focuses on commonly held views about the sexual and gender-related characteristics of LGBT+ individuals. Items in this factor assess beliefs related to masculinity, such as the perception that homosexual men are less masculine than heterosexual men (item 4). The factor also includes beliefs about sexual behavior, including the idea that LGBT+ individuals have higher sexual desire compared to heterosexual people (item 6), and that they frequently talk about sex (item 7). Higher scores in this scale reflect a more stereotyped view of LGBT+ individuals.

Each factor provides specific insights into the various attitudes being assessed by the scale. It is important to note that the questionnaire should be used as separate scales, with each factor evaluated independently, that is, without an overall score.

One of the key strengths of this study is the development of a culturally relevant instrument for measuring attitudes toward gender and sexual diversity, which can be particularly useful in Latin American populations. The scale provides a tool for researchers and practitioners to assess specific dimensions of attitudes, either positive or negative. Additionally, the collaborative nature of this research, which involved experts from several Latin American countries—including Ecuador, Argentina, Mexico, and Brazil—ensures that the instrument is not only regionally relevant but also applicable across diverse cultural settings. This cross-country involvement enriches the validity and adaptability of the scale, making it a valuable resource for future research and intervention programs focused on gender and sexual diversity issues in Latin America.

In comparison to previously developed instruments that often focus primarily on homophobia or specific subsets of LGBT+ identities (e.g., Quiles del Castillo et al., 2003; Hill and Willoughby, 2005; Costa et al., 2015), our scale offers a more comprehensive assessment of attitudes toward gender and sexual diversity, incorporating multiple dimensions such as Social Coexistence, Moral and Pathological Views, and Stereotypes. Unlike single-dimension scales that capture only negative or narrowly defined attitudes, the three-factor structure of our instrument enables a more specific and detailed evaluation of beliefs and perceptions. Moreover, by grounding item content in explicit regional contexts—where cultural values, language patterns, and social realities may differ from those usually captured in English-speaking measures—this scale fills a critical gap. Thus, its uniqueness lies in addressing the intersectionality of diverse identities, accommodating country-specific social climates, and providing robust, evidence-based psychometric properties that enable researchers and practitioners in Latin America to detect a broader spectrum of attitudes toward gender and sexual diversity.

A notable limitation of this study is that the sample was restricted to psychology students, which may not fully represent the attitudes of the general population. Psychology students, given their exposure to discussions around mental health, inclusivity, and diversity, may be more open and accepting of LGBT+ individuals compared to other segments of society. This could result in a bias toward more positive attitudes in our findings, making it difficult to generalize the results to the wider population, where attitudes may be more varied and potentially less accepting. Future research should aim to extend the use of this scale to a broader range of participants, including individuals from different educational backgrounds, professions, and social environments, to gain a more comprehensive understanding of attitudes toward gender and sexual diversity across society.

## Conclusions

The development of this scale represents an important step forward in understanding the complexities of attitudes toward gender and sexual diversity in Latin America. The scale offers a reliable and valid tool for assessing these attitudes across multiple dimensions, providing valuable insights for researchers, educators, and policymakers. However, further testing of this instrument in broader populations and in different Latin American countries would allow to ensure its applicability and relevance across diverse cultural and social contexts. Expanding its use will not only enhance the generalizability of the findings but also contribute to a more comprehensive understanding of the factors influencing attitudes toward gender and sexual diversity throughout the region. By doing so, this scale can help inform more targeted interventions and policies aimed at promoting inclusivity and reducing prejudice.

## Data Availability

The raw data supporting the conclusions of this article will be made available by the authors, without undue reservation.
